# Bioinformatics analysis of the clinical value and potential mechanisms of AHNAK2 in papillary thyroid carcinoma

**DOI:** 10.18632/aging.103645

**Published:** 2020-09-23

**Authors:** Zhenyu Xie, Yu Lun, Xin Li, Yuzhen He, Song Wu, Shiyue Wang, Jianjian Sun, Yuchen He, Jian Zhang

**Affiliations:** 1 Department of Vascular and Thyroid Surgery, The First Hospital, China Medical University, Shenyang, China

**Keywords:** AHNAK2, immune infiltration, papillary thyroid carcinoma, diagnostic biomarker, prognostic biomarker

## Abstract

Background: *AHNAK2* has been recently reported as a biomarker in many cancers. However, a systematic investigation of *AHNAK2* in papillary thyroid carcinoma (PTC) has not been conducted.

Results: *AHNAK2* is overexpressed in PTC tissues and could be an independent prognostic factor. *AHNAK2* expression was significantly high in patients with advanced stage, advanced T classification, lymph node metastasis, increased *BRAF* mutations and decreased *RAS* mutations. Cell adhesion-, cell junction-, and immune-related pathways were the most frequently noted in gene set enrichment analysis. *AHNAK2* expression in PTC was positively correlated with immune infiltration and negatively correlated with *AHNAK2* methylation. *AHNAK2* expression was significantly positively correlated with tumor progression and poor overall survival (OS) in pan-cancer patients.

Conclusions: *AHNAK2* is a good biomarker for the diagnosis and prognosis of PTC. *AHNAK2* may promote thyroid cancer progression through cell adhesion-, cell junction-, and immune-related pathways. Methylation may act as an upstream regulator to inhibit the expression and biological function of *AHNAK2*. Additionally, *AHNAK2* has broad prognostic value in pan-cancer.

Methods: Based on The Cancer Genome Atlas (TCGA) data, we screened *AHNAK2*-related genes through weighted gene coexpression network analysis and explored the clinical value and the potential mechanism of *AHNAK2* in PTC by multiomics analysis.

## INTRODUCTION

Thyroid cancer (TC) is the fifth most common cancer among women in the United States, with an estimated 52,890 new TC cases nationwide in 2020 [1], and its incidence is increasing [2, 3]. Papillary thyroid carcinoma (PTC) is the most common subtype of TC, comprising approximately 80-85% of all TCs [4, 5]. Although most well-differentiated PTC patients have an excellent overall prognosis (the 5-year survival rate of such patients is greater than 97%) [4], PTC recurrence and metastasis still hinder clinical management and prognosis in certain patients. A study with a median follow-up of 27 years also reported that 28% of PTC patients had recurrent disease, and 9% of patients died from PTC [6]. Additionally, overdiagnosis and overtreatment are common problems associated with indolent diseases. The screening and identification of indolent TC and the treatment of these overdiagnosed cancers can increase the risk of injury to patients [7, 8]. Therefore, the use of novel and sensitive biomarkers to effectively identify specific PTC patients and provide personalized treatment has become an urgent need.

*AHNAK* nucleoprotein 2 (*AHNAK2*) is a giant protein (600 kDa) with a PDZ domain that was first detected in 2004 [9, 10]. *AHNAK2* was recently reported as a biomarker for the diagnosis and prognosis of pancreatic ductal adenocarcinoma (PDAC) [11–13], clear cell renal cell carcinoma (ccRCC) [14], thymic carcinoma [15], bladder cancer [16], gastric cancer [17], and uveal melanoma (UM) [18]. Although the biological function of *AHNAK2* in cancer remains unclear, some progress has been made regarding its mechanism in tumors. *AHNAK2* is a critical element of the stress-induced FGF1 export pathway [19]. Li et al. [18] reported that *AHNAK2* may play a role in promoting the proliferation and migration of UM cells by regulating the PI3K signaling pathway. Wang et al. [14] also found that *AHNAK2* is a target gene of HIF1α, which mediates epithelial-mesenchymal transition (EMT) and stem cell characteristics driven by the hypoxia pathway, thereby promoting the progression of ccRCC.

The purpose of this study was to explore the clinical value and potential mechanism of *AHNAK2* in PTC. Based on the bioinformatics method, we conducted a systematic multiomics study of *AHNAK2* in PTC. Multiomics single-gene studies on the candidate oncogene *AHNAK2* have enriched our understanding of the molecular function of *AHNAK2* and have provided a new piece of the puzzle to the mechanism of cancer development and a basis for subsequent research on *AHNAK2*. The significant diagnostic and prognostic value of *AHNAK2* may also be used in clinical practice to assist doctors in the diagnosis and treatment of PTC patients.

## RESULTS

### Diagnostic value of AHNAK2

**Figure 1:**
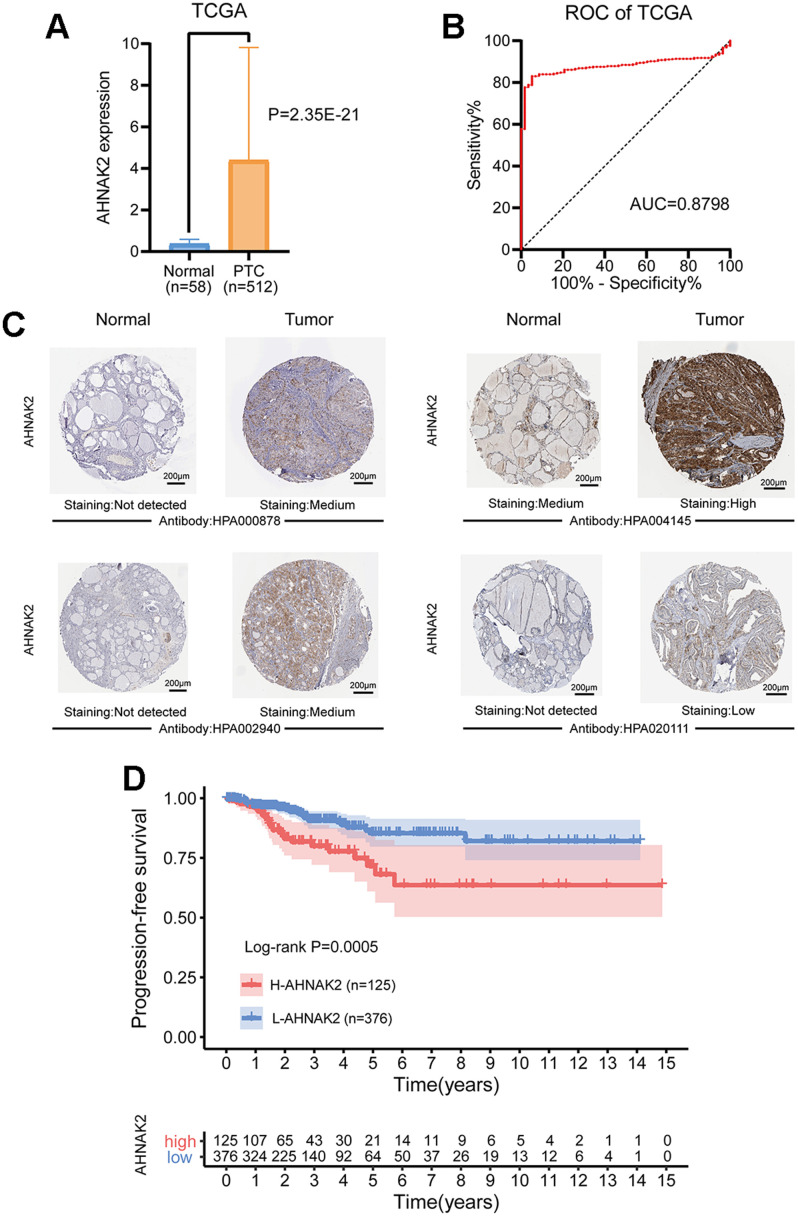
**Diagnostic and prognostic value of *AHNAK2* in PTC.** (**A**) Comparison of *AHNAK2* mRNA expression levels between PTC and normal tissues. (**B**) Diagnostic efficacy of the ROC curve of *AHNAK2*. (**C**) Comparison of immunohistochemistry images of *AHNAK2* between PTC and normal thyroid tissues with four different *AHNAK2* antibodies (HPA000878, HPA004145, HPA002940 and HPA020111) based on the Human Protein Atlas. (**D**) K-M survival analysis was performed to determine differences in PFS between the H-AHNAK2 and L-AHNAK2 groups.

The mRNA expression level of *AHNAK2* was significantly higher in PTC tissues than in normal tissues (P<0.0001) (Figure 1A). By performing receiver operating characteristic (ROC) curve analysis to discriminate PTC from normal thyroid tissues, we found that the area under the curve (AUC) of the *AHNAK2* expression level was 0.88, suggesting that it could be a good diagnostic biomarker (Figure 1B).

Additionally, immunohistochemistry performed with four different *AHNAK2* antibodies showed deeper staining levels in PTC tissues than in normal thyroid tissues, suggesting higher protein expression in PTC (Figure 1C).

### Prognostic value of AHNAK2

Kaplan-Meier (K-M) survival analysis showed that the high *AHNAK2* (H-AHNAK2) group was associated with worse progression-free survival (PFS) than the low *AHNAK2* (L-AHNAK2) group (P=0.0005) (Figure 1D). Multivariate Cox regression analysis suggested that *AHNAK2* could become an independent predictor after adjusting for other parameters, including age, gender, American Joint Committee on Cancer (AJCC) stage, BRAF mutations, RAS mutations and tumor mutation burden (TMB) (Table 1) (PFS as the ending indicator). In summary, *AHNAK2* could be an important tool for distinguishing PTC patient clinical outcomes.

**Table 1:** Univariate and multivariate regression analyses of PTC.

**Variables**	**Univariate analysis**		**Multivariate analysis**	
	**Hazard ratio (95%CI)**	***P* value**	**Hazard ratio (95%CI)**	***P* value**
Age	1.035(1.017−1.053)	<0.001	1.020(0.994−1.046)	0.132
Gender (male/female)	1.398(0.771−2.536)	0.270	1.256(0.676−2.335)	0.471
Stage	1.739(1.367−2.212)	<0.001	1.402(1.027−1.915)	0.033
BRAF mutations	1.055(0.591−1.882)	0.857	1.040(0.484−2.237)	0.920
RAS mutations	1.940(0.938−4.013)	0.074	3.356(1.277−8.818)	0.014
TMB	2.963(1.636−5.367)	<0.001	1.092(0.457−2.609)	0.843
AHNAK2	1.061(1.025−1.097)	<0.001	1.074(1.031−1.120)	<0.001

### Clinical value of AHNAK2

We analyzed the relationships between *AHNAK2* and clinical parameters, including age, gender, stage, metastasis, N classification, T classification, pathologic type, and mutations in *BRAF* and *RAS* (Table 2, Supplementary Figure 1). *AHNAK2* expression was significantly high in patients with an advanced stage, an advanced T classification, lymph node metastasis, increased *BRAF* mutations, decreased *RAS* mutations, and lower follicular PTC and higher tall-cell PTC proportions.

**Table 2:** Comparison of clinical parameters between the L-AHNAK2 and H-AHNAK2 groups in PTC.

**Clinical parameters**	**L-AHNAK2 (n=376, %)**	**H-AHNAK2 (n=125, %)**	***P*-value**
**Age(y)**			
<55	257(68.4)	77(61.6)	0.165
≥55	119(31.6)	48(38.4)	
**Gender**			
Female	272(72.3)	94(75.2)	0.532
Male	135(27.7)	31(24.8)	
**Stage**			
I	224(59.9)	57(45.6)	<0.001
II	46(12.3)	6(4.8)	
III	70(18.7)	41(32.8)	
IV	34(9.1)	21(16.8)	
NA	2	0	
**Metastasis**			
M0	209(97.7)	73(94.8)	0.214
M1	5(2.3)	4(5.2)	
NA	162	48	
**N classification**			
N0	186(55.5)	43(37.1)	0.001
N1	149(44.5)	73(62.9)	
NA	41	9	
**T classification**			
T1	120(32.1)	22(17.6)	<0.001
T2	134(35.8)	30(24.0)	
T3	111(29.7)	59(47.2)	
T4	9(2.4)	14(11.2)	
NA	2	0	
**Pathologic type**			
Classical	254(67.6)	101(80.8)	<0.001
Follicular	100(26.6)	1(0.8)	
Tall Cell	13(3.5)	23(18.4)	
Other	9(2.4)	0(0.0)	
**BRAF**			
Wild-type	181(50.4)	14(11.5)	<0.001
Mutated	178(49.6)	108(88.5)	
NA	17	3	
**RAS**			
Wild-type	299(83.3)	122(100)	<0.001
Mutated	60(16.7)	0(0.0)	
NA	17	3	

Interestingly, in the study of the relationship between *AHNAK2* expression and gene mutations in PTC (Supplementary Figure 1A), we found that *AHNAK2* expression was positively correlated with BRAF mutations (Supplementary Figure 1B) and negatively correlated with RAS (NRAS, HRAS and KRAS) mutations (Supplementary Figure 1C–1E).

### Screening modules and genes related to AHNAK2

**Figure 2:**
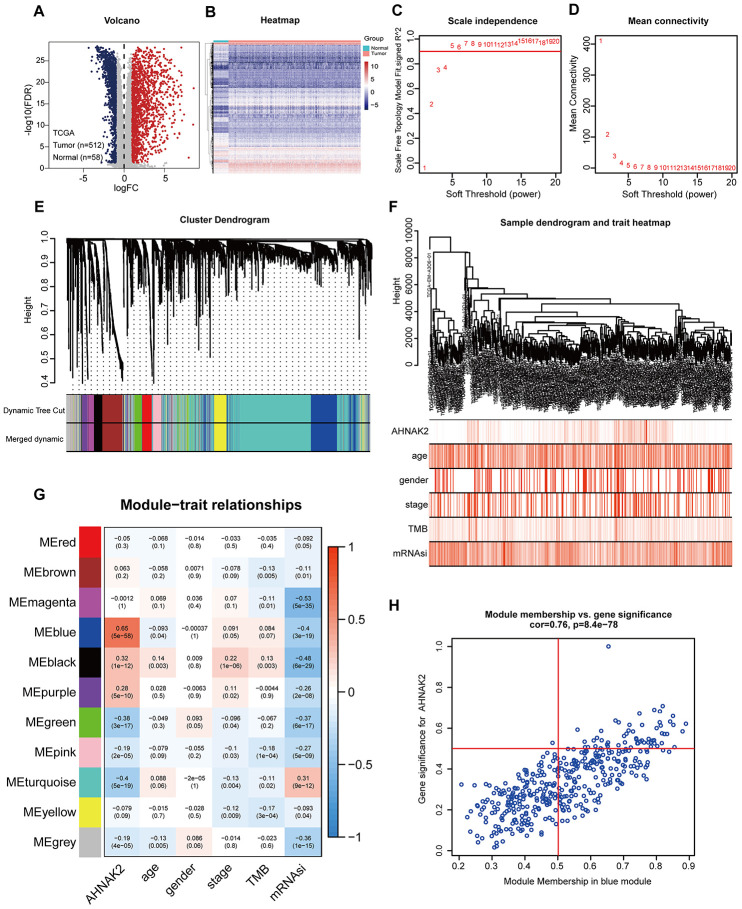
**Screening for modules and genes related to *AHNAK2* in PTC.** (**A**) Volcano plot of DEGs between PTC and adjacent tissues in TCGA data. (**B**) Heatmap of DEGs between PTC and adjacent tissues in TCGA data. (**C**) Calculation of the scale-free fit index of various soft-thresholding powers (β). (**D**) Analysis of the mean connectivity of various soft-thresholding powers (β). (**E**) Clustering dendrogram of 475 PTC patients. (**F**) A total of 2696 DEGs were clustered based on the dissimilarity measure (1-TOM) and were divided into 11 modules. (**G**) A correlation heatmap between module eigengenes and clinical parameters (*AHNAK2* was used as the main research object) of PTC. (**H**) Scatter plot of blue module eigengenes.

To construct a weighted coexpression network and screen for modules and genes related to *AHNAK2*, 2696 differentially expressed genes (DEGs) between PTC and normal tissues from The Cancer Genome Atlas (TCGA) were selected and subjected to weighted gene coexpression network analysis (WGCNA) (475 patients with complete clinical information were selected) (Figure 2A and 2B). After a series of adjustments for WGCNA parameters, the DEGs were divided into 11 modules by average linkage hierarchical clustering (Figure 2C–2F). The blue module, which contains 407 genes, exhibited the highest correlation with *AHNAK2* expression (Pearson’s correlation coefficient =0.65, P<0.0001) (Figure 2G). Fifty-seven genes in the blue module were selected as hub genes (absolute module membership [MM] > 0.5 and absolute gene significance [GS] > 0.5) (Figure 2H).

### Analysis of the potential mechanism of AHNAK2

**Figure 3:**
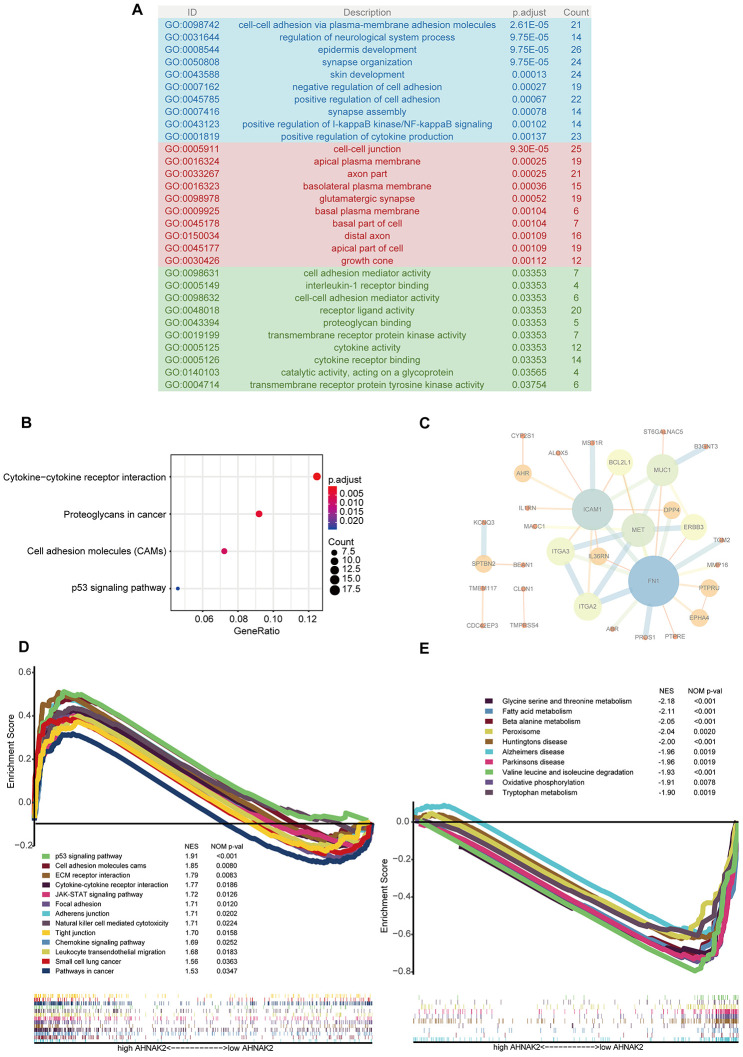
**Potential mechanisms of *AHNAK2* in PTC.** (**A**) GO analysis of 407 blue module eigengenes; the blue, red and green areas represent cellular components, biological processes and molecular functions, respectively. (**B**) KEGG analysis of blue module eigengenes: the four most significantly enriched pathways. (**C**) PPIs of hub genes. (**D**) Enriched pathways in the H-AHNAK2 group based on GSEA. (**E**) Enriched pathways in the L-AHNAK2 group based on GSEA.

Cell adhesion- and cell junction-related pathways were the most frequently noted pathways in the functional enrichment analysis of the 407 blue module genes. “Cell-cell adhesion via plasma membrane adhesion molecules”, “cell-cell junction”, and “cell adhesion mediator activity” were the most significant Gene Ontology (GO) terms for cellular components, biological processes and molecular functions, respectively (Figure 3A). “Cytokine−cytokine receptor interaction” was the most significant pathway in the Kyoto Encyclopedia of Genes and Genomes (KEGG) analysis (Figure 3B). In addition, we constructed a protein–protein interaction (PPI) network with the 57 hub genes and found that *ICAM1* and *FN1* play key roles (Figure 3C).

Additionally, gene set enrichment analysis (GSEA) was used to identify the mechanism and functional differences between the L-AHNAK2 group and H-AHNAK2 group. The H-AHNAK2 group was enriched in the following terms: 1. Cell adhesion- and cell junction-related pathways: cell adhesion molecules, focal adhesion, adherens junction, and tight junction; 2. Immune-related pathways: cytokine-cytokine receptor interaction, JAK-STAT signaling pathway, natural killer cell-mediated cytotoxicity, chemokine signaling pathway, and leukocyte transendothelial migration; and 3. Cancer-related pathways: p53 signaling pathway, small cell lung cancer, and pathways in cancer (Figure 3D). Conversely, the L-AHNAK2 group was enriched in metabolism-related pathways: glycine serine and threonine metabolism, fatty acid metabolism, beta alanine metabolism, peroxisome, valine leucine and isoleucine degradation, oxidative phosphorylation, and tryptophan metabolism (Figure 3E).

### Further analysis of the relationship between AHNAK2 and immune infiltration

**Figure 4:**
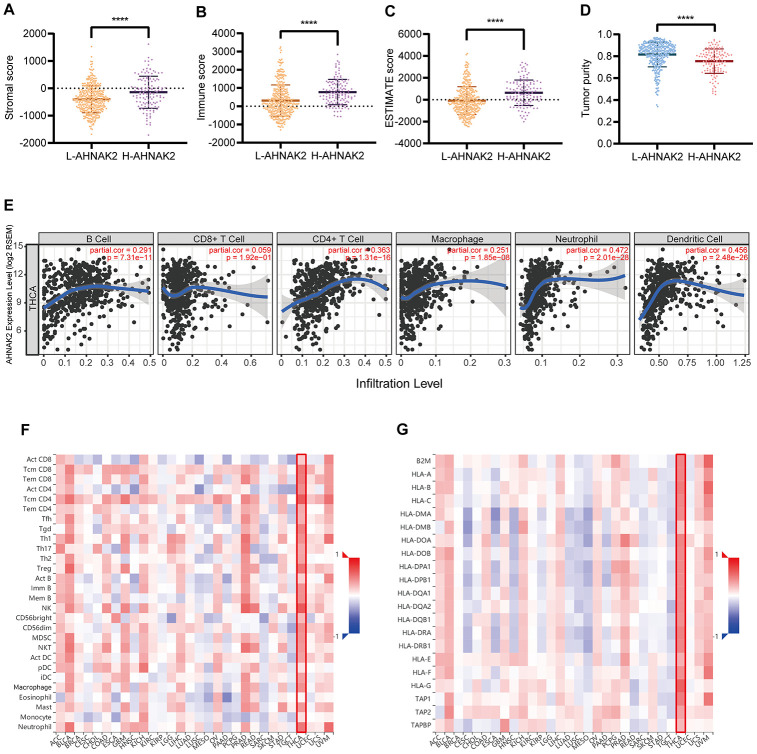
***AHNAK2* is closely related to immunity in PTC.** Comparison of (**A**) stromal scores, (**B**) immune scores, (**C**) ESTIMATE scores, and (**D**) tumor purity between the L-AHNAK2 and H-AHNAK2 groups based on ESTIMATE. (**E**) TIMER analysis of purity-corrected partial Spearman’s correlation between the expression of *AHNAK2* and six immune cells in THCA. (**F**) Correlation analysis between the expression of *AHNAK2* and 28 types of TILs across human cancers by TISIDB. (**G**) Correlation analysis between the expression of *AHNAK2* and MHCs across human cancers by TISIDB. *P<0.05, ** P<0.01, *** P<0.001, **** P<0.0001 here and in the following figures.

The results of the ESTIMATE analysis suggested that the H-AHNAK2 group had a higher immune score and stromal score than the L-AHNAK2 group, while the tumor purity score was lower (Figure 4A–4D). These results indicate that the proportions of immune cells and stromal cells are higher, and that the proportion of tumor cells is lower in the H-AHNAK2 group than in the L-AHNAK2 group. The TIMER analysis showed that *AHNAK2* has significant positive correlations with B cells, CD4^+^ T cells, macrophages, neutrophils and dendritic cells (DCs) in PTC (Figure 4E). The results of the TISIDB analysis suggested that *AHNAK2* has positive correlations with 28 tumor-infiltrating lymphocyte (TIL) types (Figure 4F) and human leukocyte antigens (HLAs) (Figure 4G) across human cancers, which is particularly significant in THCA (papillary thyroid carcinoma in TCGA).

### Analysis of AHNAK2 methylation in PTC

**Figure 5:**
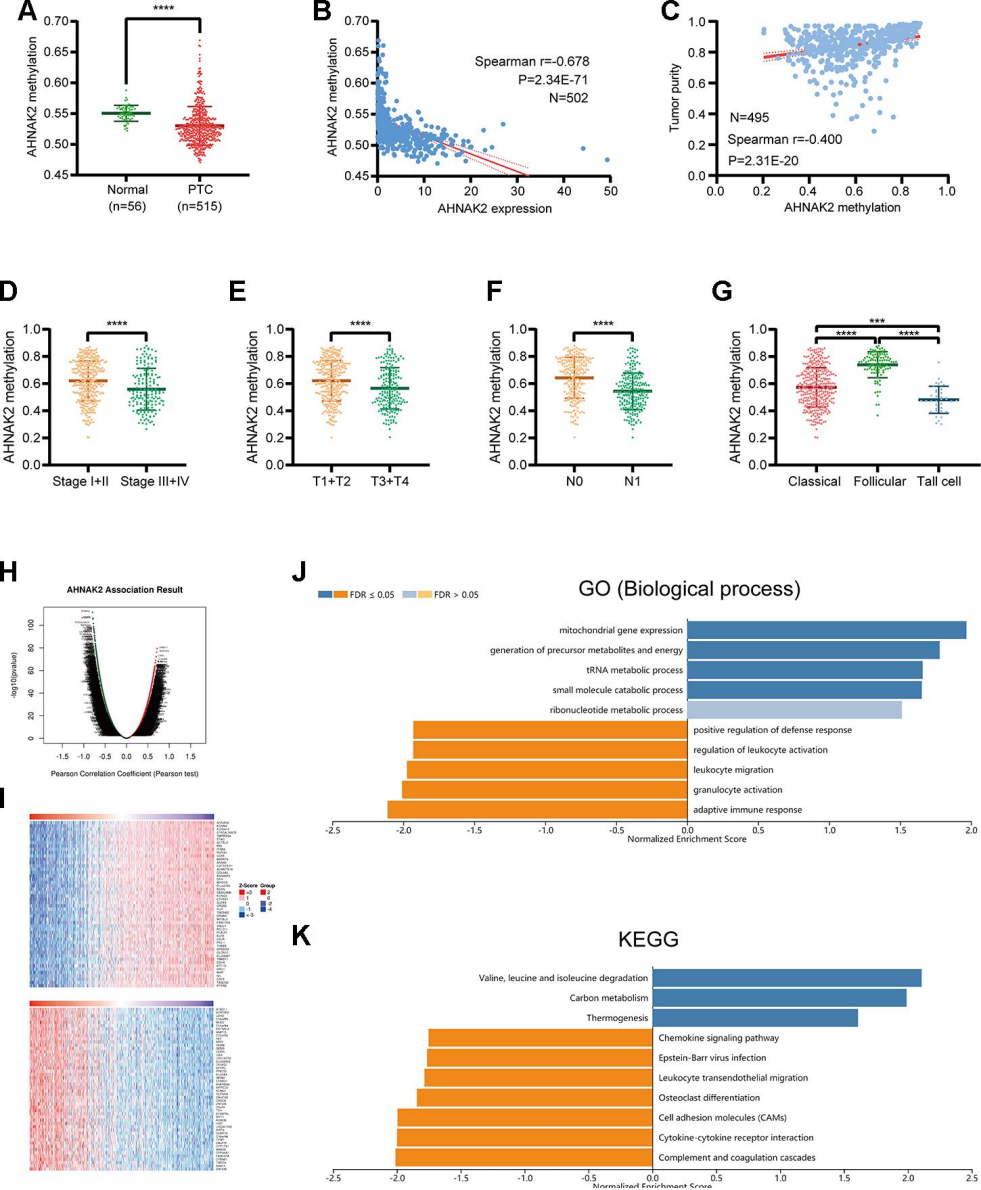
**Analysis of *AHNAK2* methylation in PTC.** (**A**) *AHNAK2* methylation levels were compared between PTC and normal tissues. (**B**) Correlation analysis between *AHNAK2* methylation and *AHNAK2* expression in PTC. (**C**) Correlation analysis between *AHNAK2* methylation and tumor purity in PTC based on LinkedOmics**.** *AHNAK2* methylation levels among PTC patients with different (**D**) stages, (**E**) T classifications, (**F**) N classifications, and (**G**) histological types. (**H**) *AHNAK2* methylation was highly correlated with genes identified by the Pearson test in the THCA cohort based on LinkedOmics. (**I**) Heatmaps of the top 50 genes positively and negatively correlated with *AHNAK2* in TCHA based on LinkedOmics. (**J**) Significantly enriched GO biological process analysis and (**K**) KEGG pathways of *AHNAK2* methylation in the THCA cohort.

Our data show that the methylation level of *AHNAK2* in PTC tissues is significantly lower than that in normal tissues (Figure 5A). A significant negative correlation was identified between the methylation level of *AHNAK2* and the expression level of *AHNAK2* (Spearman r=-0.678, P<0.0001) (Figure 5B). Among the DNA methylation sites of *AHNAK2*, cg06799735, cg01513078, cg11138227 and cg23385208 had the most significant negative correlations with the *AHNAK2* expression level (Table 3).

**Table 3:** Spearman correlations between *AHNAK2* methylation sites and *AHNAK2* expression.

**Methylation site**	**Correlation coefficient**	***P* value**
cg06799735	-0.823	1.16E-124
cg01513078	-0.765	1.60E-97
cg11138227	-0.653	2.33E-62
cg23385208	-0.507	3.44E-34
cg01412886	-0.252	9.76E-09
cg09796640	-0.252	1.08E-08
cg01987417	-0.245	2.52E-08
cg06903818	-0.189	2.03E-05
cg07828276	-0.178	5.81E-05
cg04908382	0.174	8.61E-05
cg26082174	-0.169	1.39E-04
cg02048807	-0.132	0.003
cg21868779	-0.118	0.008
cg07450030	-0.097	0.031
cg14976660	-0.096	0.032
cg02719508	-0.086	0.055
cg18958585	-0.078	0.080
cg13108289	0.073	0.103
cg01238264	0.048	0.280
cg10207355	-0.045	0.315
cg05376485	-0.044	0.324
cg14046988	0.036	0.427
cg04725566	0.035	0.427
cg19901468	0.03	0.504
cg07071690	-0.026	0.564
cg03006175	-0.017	0.699
cg16396153	-0.007	0.875
cg17340268	-0.005	0.910
cg27161497	-0.005	0.915
cg16097532	-0.002	0.969
cg13599499	NA	NA

Moreover, we found significant connections between *AHNAK2* methylation and clinical parameters such as tumor purity, stage, T classification, N classification and histological type (Figure 5C–5G).

To understand the biological significance of *AHNAK2* methylation in THCA, a functional module of LinkedOmics was used to examine *AHNAK2* coexpression pattern in the THCA cohort. Based on RNAseq, we screened 9,944 genes related to *AHNAK2* methylation (false discovery rate (FDR) <0.01) (Figure 5H). The top 50 significant genes that were positively and negatively correlated with *AHNAK2* methylation are presented as two heatmaps in Figure 5I. The GO (biological process) analysis results derived by GSEA were significant; the results indicated that *AHNAK2* methylation coexpressed genes participate primarily in metabolism-related pathways, while genes related to activities such as the adaptive immune response, granulocyte activation, leukocyte migration, regulation of leukocyte activation, and positive regulation of the defense response were inhibited. KEGG pathway analysis also showed that genes related to the complement and coagulation cascades, cytokine-cytokine receptor interaction, cell adhesion molecules, and Epstein–Barr virus infection, among other pathways, were inhibited. These results illustrate the extensive effects of *AHNAK2* methylation on transcriptome and immune-related pathways.

### Gene expression omnibus (GEO) verification of the diagnostic value of AHNAK2

**Figure 6:**
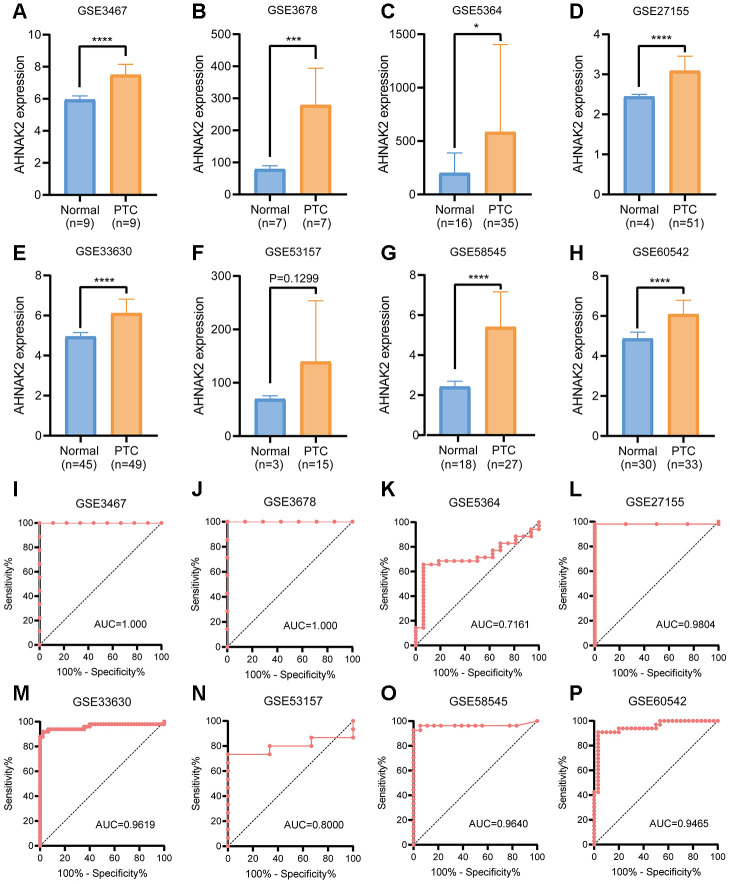
**GEO verification of the diagnostic value of *AHNAK2* in PTC.** (**A**–**H**) mRNA expression levels of *AHNAK2* in 8 GEO verification cohorts (GSE3467, GSE3678, GSE5364, GSE27155, GSE33630, GSE58545, GSE53157, and GSE60542). (**I**–**P**) ROC curves were generated to verify the diagnostic performance of *AHNAK2* expression in the verification cohorts.

By analyzing mRNA expression in 8 GEO validation cohorts (GSE3467, GSE3678, GSE5364, GSE27155, GSE33630, GSE58545, GSE53157, and GSE60542), we verified the efficacy of *AHNAK2* in distinguishing PTC from normal tissues (Figure 6A–6P).

### Generalization value of AHNAK2 in pan-cancer

**Figure 7:**
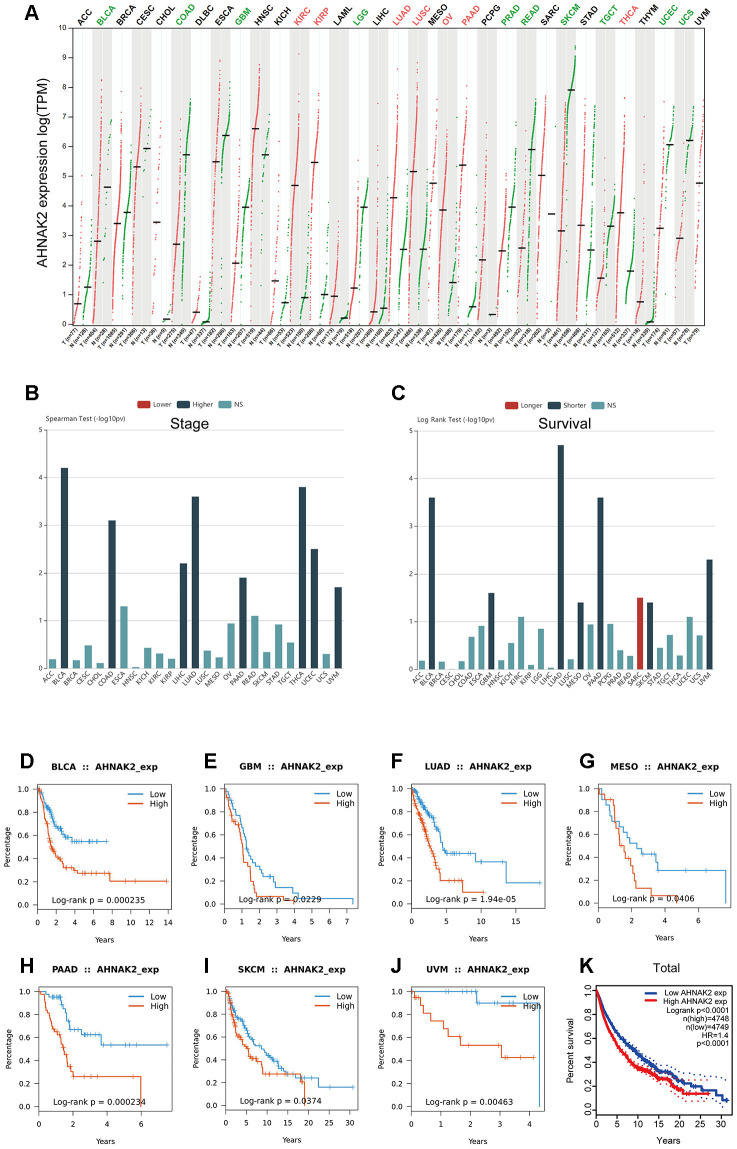
**Generalization value of *AHNAK2* in pan-cancer.** (**A**) Comparison of *AHNAK2* mRNA expression between pan-cancer cancerous and paracancerous tissues based on GEPIA. (**B**) Associations between *AHNAK2* expression and stage across human cancers based on TISIDB. (**C**) Associations between *AHNAK2* expression and overall survival across human cancers based on TISIDB. K-M survival analysis of (**D**) BLCA, (**E**) GBM, (**F**) LUAD, (**G**) MESO, (**H**) PAAD, (**I**) SKCM, (**J**) UVM, and (**K**) pan-cancer in the low *AHNAK2* and high *AHNAK2* groups based on TISIDB and GEPIA. NS: no significance.

To investigate whether *AHNAK2* has broad value, we performed a series of studies on *AHNAK2* across all cancers. Gene Expression Profiling Interactive Analysis (GEPIA) showed that *AHNAK2* expression status varies in different cancers (Figure 7A). TISIDB showed that the high expression of *AHNAK2* in pan-cancer tended to be accompanied by an advanced tumor stage (Figure 7B) and short overall survival (OS) (Figure 7C). K-M survival analysis showed that the high *AHNAK2* group had significant associations with short OS in bladder urothelial carcinoma (BLCA), glioblastoma multiforme (GBM), lung adenocarcinoma (LUAD), mesothelioma (MESO), pancreatic adenocarcinoma (PAAD), skin cutaneous melanoma (SKCM) and uveal melanoma (UVM) (Figure 7D–7J). *AHNAK2* is an extremely significant gene in the survival statistics of a large number of pan-cancer samples (N = 9497, HR = 1.4, P<0.0001) (Figure 7K).

## DISCUSSION

Our research suggests that *AHNAK2* can be a biomarker for the diagnosis and prognosis of PTC. *AHNAK2* functions mainly in cell adhesion-, cell junction-, and immune-related pathways. *AHNAK2* also has extensive prognostic value in pan-cancer.

We previously found that *AHNAK2* has a significant negative correlation with the prognosis of PTC and used *AHNAK2* as one of the model genes for incorporation into prognostic models. Using this preliminary finding, we conducted a systematic study on the role of *AHNAK2* in PTC. In recent years, many studies have reported that *AHNAK2* could be a biomarker for the diagnosis and prognosis of PDAC, ccRCC, thymic carcinogenesis, bladder cancer, gastric cancer, and UM [11–18]. Kim et al. [20] also noted that high CXCL16 expression combined with the high expression levels of related genes (e.g., AHNAK2 and THBS2) was associated with poor recurrence-free survival in PTC patients. However, a systematic investigation of *AHNAK2* in PTC has not been conducted. Our research fills the knowledge gap in the study of *AHNAK2* in PTC and reveals the potential mechanism of *AHNAK2* in PTC. This study also provides strong support for *AHNAK2* as a prognostic biomarker for pan-cancer.

*AHNAK2* has been described as a diagnostic biomarker in PDAC [11, 12], thymic carcinoma [15], and bladder cancer [16]. We found that overexpression of *AHNAK2* in PTC tissues can be used to distinguish between PTC and normal tissues. We further verified the outstanding clinical diagnostic efficacy of *AHNAK2* in 8 GEO verification cohorts.

*AHNAK2* has been described as a prognostic biomarker in ccRCC [14] and PDAC [11, 13]. Our results show that *AHNAK2* can be used as an independent prognostic predictor in PTC and to classify the PTC population and provide personalized treatment to reduce harm to patients caused by overtreatment. *AHNAK2* may play a role in promoting the proliferation and migration of UM cells by regulating the PI3K signaling pathway [18]. A recent study also found that *AHNAK2* is a target gene of HIF1α, which mediates EMT and stem cell characteristics driven by the hypoxia pathway, thereby promoting the progression of ccRCC [14]. *AHNAK2* expression is significantly high in patients with an advanced cancer stage, an advanced T classification, lymph node metastasis, and increased BRAF mutations, showing a positive correlation with PTC progression. These findings imply that *AHNAK2* may exert a tumor-promoting effect in PTC.

Interestingly, the two genes with the highest mutation rates in PTC [21] exhibited a notable trend: BRAF mutations were positively associated with *AHNAK2* expression, while RAS mutations were negatively correlated with *AHNAK2* expression.

Through functional enrichment analysis of the WGCNA-selected module eigengenes, *AHNAK2* was determined to be most closely related to cell adhesion and cell junction pathways and inflammatory pathways in PTC. The GSEA results also revealed that *AHNAK2* may promote progression in PTC through cell adhesion-, cell junction-, immune-, and cancer-related pathways.

Previous studies have noted that *AHNAK2*, as a scaffolding protein, is associated with voltage-gated calcium channels [9], and calcium plays a key role in the regulation of cell adhesion and cell junctions [22, 23]. Thus, these results can help explain the enrichment of cell adhesion- and cell junction-related pathways in our study.

Related reports of *AHNAK2* in systemic lupus erythematosus (SLE) indicate its potential link with immunity [24, 25]. *AHNAK2* is a critical element of the stress-induced FGF1 export pathway [19]. FGF1 is a nonclassically released growth factor that regulates carcinogenesis, angiogenesis and inflammation in tumors. Based on the results of our previous research and functional analysis, we further explored the relationship between *AHNAK2* and immunity in PTC. Through ESTIMATE analysis, we found that high *AHNAK2* expression indicates high degrees of immune and stromal cell infiltration and low tumor purity in the PTC microenvironment. The TIMER analysis showed that *AHNAK2* is positively correlated with immune infiltration. The correlation analysis between *AHNAK2* and 28 immune cell types in TISIDB showed that the entire immune system and *AHNAK2* are positively correlated in PTC. The above results suggest that *AHNAK2* may promote the occurrence and development of PTC through immune-related pathways.

*AHNAK2* is uniquely monomethylated by the protein lysine monomethyltransferase (SMYD2) and is involved in human esophageal squamous carcinoma (ESCC) [26]. Ohmura et al. [27] reported that the methylation of *AHNAK2* is associated with chemotherapy resistance in Epstein–Barr virus (EBV)-associated gastric cancer. Our research on PTC epigenetics has also shown that methylation may act as an upstream regulatory mechanism of *AHNAK2* expression to inhibit its biological function.

Our study found that although the expression levels of *AHNAK2* differ in various tumors, they are significantly positively correlated with tumor progression and short OS in pan-cancer patients, indicating that *AHNAK2* also has universal prognostic value in pan-cancer.

Our study found that *AHNAK2* can be used as a diagnostic and prognostic biomarker in PTC. *AHNAK2* is believed to be able to identify critical cases of PTC and avoid overdiagnosis. The prognostic value of *AHNAK2* should assist doctors in providing personalized treatment to patients and avoiding harm to patients caused by overtreatment. Studies in pan-carcinomas have shown the broad clinical application value of *AHNAK2*. Multiomics single-gene studies on the candidate oncogene *AHNAK2* have enriched our understanding of the molecular function of *AHNAK2* and have provided a new piece of the puzzle to the mechanism of cancer development and a basis for subsequent research on *AHNAK2*.

However, this study has some limitations. First, because TCGA is the only public database with sufficient data on both PTC gene expression and prognosis, we did not use other independent cohorts to verify the prognostic value of *AHNAK2* in PTC. Second, the molecular functions of *AHNAK2* in PTC were analyzed through bioinformatics, and the conclusions have not been further confirmed experimentally. However, related experimental research is ongoing.

*AHNAK2* can be used as a diagnostic and prognostic biomarker in PTC. *AHNAK2* may promote the progression of PTC through cell adhesion-, cell junction-, and immune-related pathways, and methylation may serve as an upstream regulator to inhibit the expression and biological functions of *AHNAK2*. *AHNAK2* also has extensive prognostic value in pan-cancer. Research on the cancer candidate gene *AHNAK2* will help us understand the development and prognosis of cancer and provide a piece of the puzzle to overcome cancer.

## MATERIALS AND METHODS

### Materials

The TCGA THCA dataset, which contains 58 normal thyroid samples (N) and 512 PTC samples (T), was selected as the discovery cohort. The normalized level-three RNA-seq data (FPKM), simple nucleotide variation data (VarScan) and DNA methylation data were downloaded from the TCGA GDC (https://portal.gdc.cancer.gov/). The clinical information on TCGA THCA was downloaded from the University of California at Santa Cruz (UCSC) Xena browser (https://xena.ucsc.edu/).

Eight gene expression microarray datasets, each containing both PTC and normal thyroid tissue samples, were downloaded from the National Center for Biotechnology Information GEO database (http://www.ncbi.nlm.nih.gov/geo): GSE3467 (N=9, T=9), GSE3678 (N=7, T=7), GSE5364 (N=16, T=35), GSE27155 (N=4, T=51), GSE33630 (N=45, T=49), GSE58545 (N=18, T=27), GSE53157 (N=3, T=15), and GSE60542 (N=30, T=33).

### Comparison of AHNAK2 expression between THCA and normal thyroid tissues

The mRNA expression levels of *AHNAK2* in PTC and normal thyroid tissues were analyzed using TCGA data. We defined the top quarter as the H-AHNAK2 group (based on mRNA expression rank in the THCA dataset) and the remainder as the L-AHNAK2 group.

ROC curve analysis was used to evaluate the diagnostic efficacy of *AHNAK2*. The AUC followed the criteria 0.50–0.60 = fail, 0.60–0.70 = poor, 0.70–0.80 = fair, 0.80–0.90 = good and 0.90–1 = excellent.

Additionally, a direct comparison of *AHNAK2* protein expression between PTC and normal thyroid tissues was performed by analyzing immunohistochemistry images from The Human Protein Atlas (https://www.proteinatlas.org) [28].

### WGCNA reveals key modules and hub genes related to AHNAK2

First, DEGs between PTC and normal tissues in the TCGA dataset were identified with the “limma” package in R (adjusted p-value <0.05 and |log_2_FC| >1).

WGCNA aims to identify coexpressed gene modules and to explore associations between gene networks and phenotypes of interest, as well as core genes in the network [29]. We divided the DEGs into different gene modules using the “WGCNA” package in R (minModuleSize = 30).

We defined the module with the highest absolute module significance as the key module. We defined the hub genes as those that satisfied the following criteria:

1. In the key module;
2. An absolute value of MM >0.5, which represents Pearson’s correlation between the gene and the module; and
3. An absolute value of GS >0.5, which represents Pearson’s correlation between the gene and the clinical parameter.

### Analysis of the potential mechanism of AHNAK2 in PTC

### GO and KEGG enrichment analyses

The genes in the key module were subjected to GO and KEGG enrichment analyses with DAVID 6.7 (https://david-d.ncifcrf.gov/).

### PPI network analysis

The hub genes were input into the STRING database (version 11.0, https://string-db.org/) to analyze the interaction network of the hub gene-encoded proteins. The minimum required interaction score was set to 0.4 (medium confidence), and the results were visualized using Cytoscape (v3.7.2).

### GSEA

GSEA software (Version 2.0.1) (http://www.broad.mit.edu/gsea) was used to explore the potential biological function of *AHNAK2* in PTC. A normalized enrichment score (NES) ≥ 1.0, a p-value ≤ 0.05, and an FDR q-val ≤ 0.25 were used as criteria to sort the GSEA results (c2.cp.kegg.v6.2.symbols.gmt was used as the reference gene set) into the L-AHNAK2 and H-AHNAK2 groups after 1000 permutations for each analysis.

### Further analysis of the relationship between AHNAK2 and immune infiltration in PTC

### ESTIMATE

ESTIMATE [30], a method that uses gene expression signatures to infer the fractions of stromal and immune cells in tumor samples, was used to evaluate the levels of immune cell infiltration (immune score), the stromal content (stromal score), the stromal-immune comprehensive score (ESTIMATE score) and tumor purity for each THCA sample.

### TIMER

The TIMER online database [31], a web server for comprehensive analysis of tumor-infiltrating immune cells, was used to analyze and visualize associations between *AHNAK2* expression and the abundance of 6 tumor-infiltrating immune cell subtypes (B cells, CD4^+^ T cells, CD8^+^ T cells, macrophages, neutrophils, and DCs).

### TISIDB

We used TISIDB, a comprehensive repository portal for tumor-immune system interactions, to determine the Spearman correlations between *AHNAK2* expression and 28 TIL types across human cancers [32].

### Analysis of AHNAK2 methylation in PTC

Based on DNA methylation data downloaded from TCGA, we compared the methylation levels of *AHNAK2* between PTC and normal tissues. We analyzed the correlation between AHNAK2 methylation and *AHNAK2* expression in PTC. We also performed a correlation analysis between each methylation site of *AHNAK2* and *AHNAK2* expression.

LinkedOmics is an online platform for analyzing multiomics data across 32 cancer types (http://www.linkedomics.org/) [33]. We downloaded and visualized the correlation results between *AHNAK2* methylation and clinical parameters from LinkedOmics. Pearson correlations were used to analyze the *AHNAK2* methylation-induced coexpression of genes, and the results are displayed with a volcano map and a heatmap. The functional modules of LinkedOmics are based on *AHNAK2* methylation data, and GSEA was performed with data from GO (biological process) and KEGG pathway databases. We set the minimum number of genes (Size) =10 and simulations=1000 and selected a weighted set cover to reduce redundancy.

### GEO verification of the diagnostic value of AHNAK2

mRNA expression and ROC curves were used to verify the diagnostic efficacy of *AHNAK2* in 8 GEO datasets (GSE3467, GSE3678, GSE5364, GSE27155, GSE33630, GSE58545, GSE53157, and GSE60542).

### Generalization of conclusions in pan-cancer

GEPIA (http://gepia.cancer-pku.cn/) is used as a tool to generalize conclusions in pan-cancer [34]. A comparison of *AHNAK2* gene transcripts between PTC and normal thyroid tissues in pan-cancer was performed using TCGA and Genotype-Tissue Expression (GTEx) data (p-value <0.05 and |log_2_FC| >1). K-M curves in pan-cancer were generated using the cohort of TCGA data. We used TISIDB to analyze relationships between *AHNAK2* expression and tumor stage in pan-cancer, as well as associations between *AHNAK2* expression and OS.

### Statistical analysis

K-M survival analysis was performed with the "survival" package (PFS was used as the endpoint). The chi-square test was used to test differences in clinical parameters between the L-AHNAK2 and H-AHNAK2 groups. The Spearman method was used to test for correlations. The Mann-Whitney test was used to compare two groups. The log-rank method was used to calculate significant P values for survival. R (v3.6.0) and SPSS version 25.0 software programs were used for statistical processing. Data were visualized with GraphPad Prism V.8.0 software and R.

## Supplementary Material

Supplementary Figure 1

## References

[1] Siegel RL, Miller KD, Jemal A. Cancer statistics, 2020. CA Cancer J Clin. 2020; 70:7–30. 10.3322/caac.21590 31912902

[2] Cabanillas ME, McFadden DG, Durante C. Thyroid cancer. Lancet. 2016; 388:2783–95. 10.1016/S0140-6736(16)30172-6 27240885

[3] Li N, Du XL, Reitzel LR, Xu L, Sturgis EM. Impact of enhanced detection on the increase in thyroid cancer incidence in the United States: review of incidence trends by socioeconomic status within the surveillance, epidemiology, and end results registry, 1980-2008. Thyroid. 2013; 23:103–10. 10.1089/thy.2012.0392 23043274 PMC3539256

[4] Schneider DF, Chen H. New developments in the diagnosis and treatment of thyroid cancer. CA Cancer J Clin. 2013; 63:374–94. 10.3322/caac.21195 23797834 PMC3800231

[5] Fagin JA, Wells SA Jr. Biologic and clinical perspectives on thyroid cancer. N Engl J Med. 2016; 375:1054–67. 10.1056/NEJMra1501993 27626519 PMC5512163

[6] Grogan RH, Kaplan SP, Cao H, Weiss RE, Degroot LJ, Simon CA, Embia OM, Angelos P, Kaplan EL, Schechter RB. A study of recurrence and death from papillary thyroid cancer with 27 years of median follow-up. Surgery. 2013; 154:1436–46. 10.1016/j.surg.2013.07.008 24075674

[7] Lin JS, Bowles EJ, Williams SB, Morrison CC. Screening for thyroid cancer: updated evidence report and systematic review for the US preventive services task force. JAMA. 2017; 317:1888–903. 10.1001/jama.2017.056228492904

[8] Kitahara CM, Sosa JA. The changing incidence of thyroid cancer. Nat Rev Endocrinol. 2016; 12:646–53. 10.1038/nrendo.2016.110 27418023 PMC10311569

[9] Komuro A, Masuda Y, Kobayashi K, Babbitt R, Gunel M, Flavell RA, Marchesi VT. The AHNAKs are a class of giant propeller-like proteins that associate with calcium channel proteins of cardiomyocytes and other cells. Proc Natl Acad Sci USA. 2004; 101:4053–58. 10.1073/pnas.0308619101 15007166 PMC384694

[10] Han H, Kursula P. Periaxin and AHNAK nucleoprotein 2 form intertwined homodimers through domain swapping. J Biol Chem. 2014; 289:14121–31. 10.1074/jbc.M114.554816 24675079 PMC4022880

[11] Klett H, Fuellgraf H, Levit-Zerdoun E, Hussung S, Kowar S, Küsters S, Bronsert P, Werner M, Wittel U, Fritsch R, Busch H, Boerries M. Identification and validation of a diagnostic and prognostic multi-gene biomarker panel for pancreatic ductal adenocarcinoma. Front Genet. 2018; 9:108. 10.3389/fgene.2018.00108 29675033 PMC5895731

[12] Bhasin MK, Ndebele K, Bucur O, Yee EU, Otu HH, Plati J, Bullock A, Gu X, Castan E, Zhang P, Najarian R, Muraru MS, Miksad R, et al. Meta-analysis of transcriptome data identifies a novel 5-gene pancreatic adenocarcinoma classifier. Oncotarget. 2016; 7:23263–81. 10.18632/oncotarget.8139 26993610 PMC5029625

[13] Lu D, Wang J, Shi X, Yue B, Hao J. AHNAK2 is a potential prognostic biomarker in patients with PDAC. Oncotarget. 2017; 8:31775–84. 10.18632/oncotarget.15990 28423668 PMC5458247

[14] Wang M, Li X, Zhang J, Yang Q, Chen W, Jin W, Huang YR, Yang R, Gao WQ. AHNAK2 is a novel prognostic marker and oncogenic protein for clear cell renal cell carcinoma. Theranostics. 2017; 7:1100–13. 10.7150/thno.18198 28435451 PMC5399579

[15] Saito M, Fujiwara Y, Asao T, Honda T, Shimada Y, Kanai Y, Tsuta K, Kono K, Watanabe S, Ohe Y, Kohno T. The genomic and epigenomic landscape in thymic carcinoma. Carcinogenesis. 2017; 38:1084–91. 10.1093/carcin/bgx094 28968686

[16] Witzke KE, Großerueschkamp F, Jütte H, Horn M, Roghmann F, von Landenberg N, Bracht T, Kallenbach-Thieltges A, Käfferlein H, Brüning T, Schork K, Eisenacher M, Marcus K, et al. Integrated fourier transform infrared imaging and proteomics for identification of a candidate histochemical biomarker in bladder cancer. Am J Pathol. 2019; 189:619–31. 10.1016/j.ajpath.2018.11.018 30770125

[17] Zhou YY, Kang YT, Chen C, Xu FF, Wang HN, Jin R. Combination of TNM staging and pathway based risk score models in patients with gastric cancer. J Cell Biochem. 2018; 119:3608–17. 10.1002/jcb.26563 29231991

[18] Li M, Liu Y, Meng Y, Zhu Y. AHNAK nucleoprotein 2 performs a promoting role in the proliferation and migration of uveal melanoma cells. Cancer Biother Radiopharm. 2019; 34:626–33. 10.1089/cbr.2019.2778 31621397

[19] Kirov A, Kacer D, Conley BA, Vary CP, Prudovsky I. AHNAK2 participates in the stress-induced nonclassical FGF1 secretion pathway. J Cell Biochem. 2015; 116:1522–31. 10.1002/jcb.25047 25560297 PMC4697109

[20] Kim MJ, Sun HJ, Song YS, Yoo SK, Kim YA, Seo JS, Park YJ, Cho SW. CXCL16 positively correlated with M2-macrophage infiltration, enhanced angiogenesis, and poor prognosis in thyroid cancer. Sci Rep. 2019; 9:13288. 10.1038/s41598-019-49613-z 31527616 PMC6746802

[21] Rossi M, Buratto M, Tagliati F, Rossi R, Lupo S, Trasforini G, Lanza G, Franceschetti P, Bruni S, Degli Uberti E, Zatelli MC. Relevance of BRAF(V600E) mutation testing versus RAS point mutations and RET/PTC rearrangements evaluation in the diagnosis of thyroid cancer. Thyroid. 2015; 25:221–28. 10.1089/thy.2014.0338 25333496 PMC4322031

[22] Stuart RO, Sun A, Panichas M, Hebert SC, Brenner BM, Nigam SK. Critical role for intracellular calcium in tight junction biogenesis. J Cell Physiol. 1994; 159:423–33. 10.1002/jcp.1041590306 8188760

[23] Kemler R, Ozawa M, Ringwald M. Calcium-dependent cell adhesion molecules. Curr Opin Cell Biol. 1989; 1:892–97. 10.1016/0955-0674(89)90055-0 2697291

[24] Akizuki S, Ishigaki K, Kochi Y, Law SM, Matsuo K, Ohmura K, Suzuki A, Nakayama M, Iizuka Y, Koseki H, Ohara O, Hirata J, Kamatani Y, et al. PLD4 is a genetic determinant to systemic lupus erythematosus and involved in murine autoimmune phenotypes. Ann Rheum Dis. 2019; 78:509–18. 10.1136/annrheumdis-2018-214116 30679154

[25] Wen L, Zhu C, Zhu Z, Yang C, Zheng X, Liu L, Zuo X, Sheng Y, Tang H, Liang B, Zhou Y, Li P, Zhu J, et al. Exome-wide association study identifies four novel loci for systemic lupus erythematosus in han Chinese population. Ann Rheum Dis. 2018; 77:417. 10.1136/annrheumdis-2017-211823 29233832

[26] Olsen JB, Cao XJ, Han B, Chen LH, Horvath A, Richardson TI, Campbell RM, Garcia BA, Nguyen H. Quantitative profiling of the activity of protein lysine methyltransferase SMYD2 using SILAC-based proteomics. Mol Cell Proteomics. 2016; 15:892–905. 10.1074/mcp.M115.053280 26750096 PMC4813708

[27] Ohmura H, Ito M, Uchino K, Okada C, Tanishima S, Yamada Y, Momosaki S, Komoda M, Kuwayama M, Yamaguchi K, Okumura Y, Nakano M, Tsuchihashi K, et al. Methylation of drug resistance-related genes in chemotherapy-sensitive epstein-barr virus-associated gastric cancer. FEBS Open Bio. 2020; 10:147–57. 10.1002/2211-5463.12765 31736281 PMC6943226

[28] Asplund A, Edqvist PH, Schwenk JM, Pontén F. Antibodies for profiling the human proteome-the human protein atlas as a resource for cancer research. Proteomics. 2012; 12:2067–77. 10.1002/pmic.201100504 22623277

[29] Langfelder P, Horvath S. WGCNA: an R package for weighted correlation network analysis. BMC Bioinformatics. 2008; 9:559. 10.1186/1471-2105-9-559 19114008 PMC2631488

[30] Yoshihara K, Shahmoradgoli M, Martínez E, Vegesna R, Kim H, Torres-Garcia W, Treviño V, Shen H, Laird PW, Levine DA, Carter SL, Getz G, Stemke-Hale K, et al. Inferring tumour purity and stromal and immune cell admixture from expression data. Nat Commun. 2013; 4:2612. 10.1038/ncomms3612 24113773 PMC3826632

[31] Li T, Fan J, Wang B, Traugh N, Chen Q, Liu JS, Li B, Liu XS. TIMER: a web server for comprehensive analysis of tumor-infiltrating immune cells. Cancer Res. 2017; 77:e108–10. 10.1158/0008-5472.CAN-17-0307 29092952 PMC6042652

[32] Ru B, Wong CN, Tong Y, Zhong JY, Zhong SS, Wu WC, Chu KC, Wong CY, Lau CY, Chen I, Chan NW, Zhang J. TISIDB: an integrated repository portal for tumor-immune system interactions. Bioinformatics. 2019; 35:4200–02. 10.1093/bioinformatics/btz210 30903160

[33] Vasaikar SV, Straub P, Wang J, Zhang B. LinkedOmics: analyzing multi-omics data within and across 32 cancer types. Nucleic Acids Res. 2018; 46:D956–63. 10.1093/nar/gkx1090 29136207 PMC5753188

[34] Tang Z, Li C, Kang B, Gao G, Li C, Zhang Z. GEPIA: a web server for cancer and normal gene expression profiling and interactive analyses. Nucleic Acids Res. 2017; 45:W98–102. 10.1093/nar/gkx247 28407145 PMC5570223

