# Supplementary material for: Bioinformatics analysis of the clinical value and potential mechanisms of AHNAK2 in papillary thyroid carcinoma
Source: Aging (Albany NY). 2020 Sep 23;12(18):18163–80. doi: 10.18632/aging.103645 (PMC7585101; doi:10.18632/aging.103645)
Supplement: Supplementary Figure 1 [file aging-12-103645-s001..pdf]

SUPPLEMENTARY FIGURE

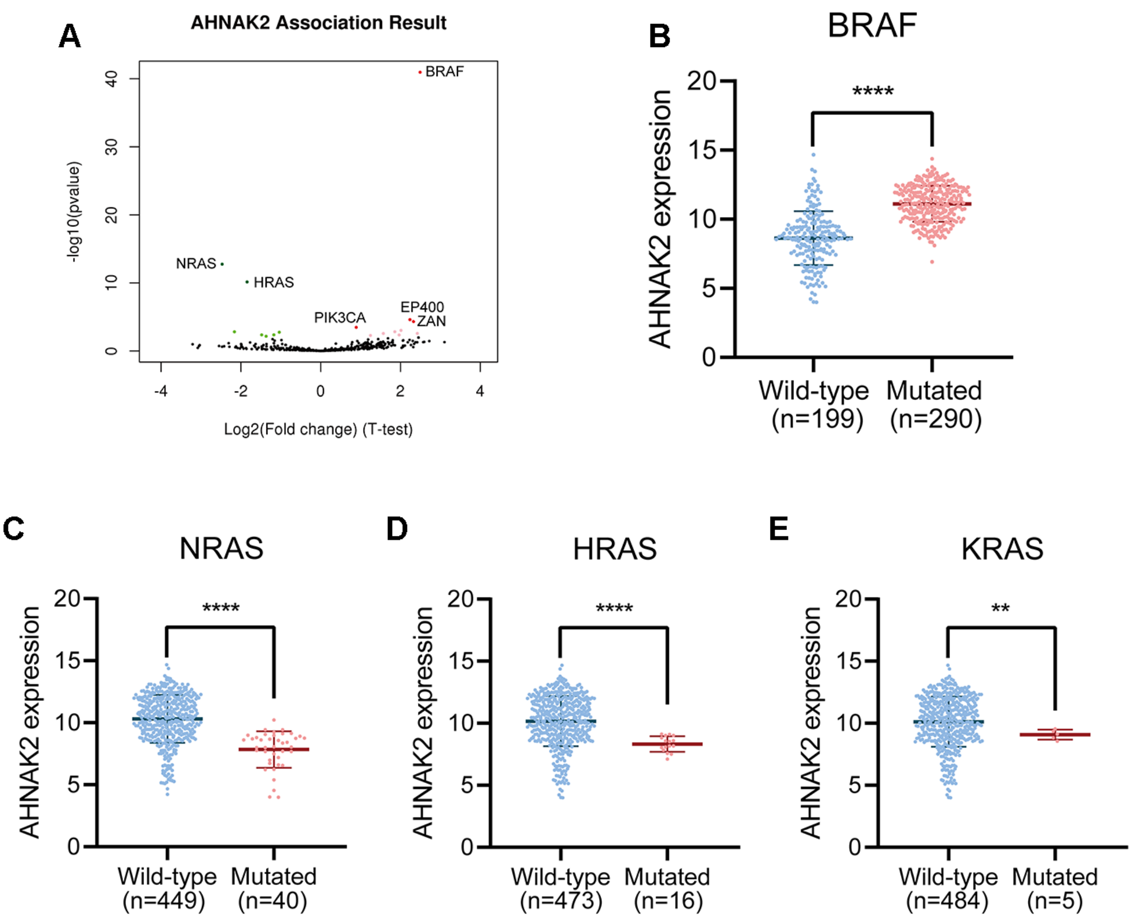

Supplementary Figure 1. Relationship between AHANK2 expression and gene mutation in PTC (LinkedOmics). (**A**) Volcano map of the relationship between ANHAK2 expression and gene mutation in THCA. (**B**) Effect of BRAF mutation, (**C**) NRAS mutation, (**D**) HRAS mutation and (**E**) KRAS mutation on ANHAK2 expression level.
